# ‘Puppy Dog Eyes’ Are Associated With Eye Movements, Not Communication

**DOI:** 10.3389/fpsyg.2021.568935

**Published:** 2021-02-17

**Authors:** Annika Bremhorst, Daniel S. Mills, Lisa Stolzlechner, Hanno Würbel, Stefanie Riemer

**Affiliations:** ^1^Division of Animal Welfare, DCR-VPHI, Vetsuisse Faculty, University of Bern, Bern, Switzerland; ^2^School of Life Sciences, University of Lincoln, Lincoln, United Kingdom; ^3^Graduate School for Cellular and Biomedical Sciences (GCB), University of Bern, Bern, Switzerland; ^4^Department of Cognitive Biology, University of Vienna, Vienna, Austria

**Keywords:** dog, DogFACS, facial expressions, inner brow raiser, AU101, puppy dog eyes, social use, signal

## Abstract

The inner brow raiser is a muscle movement that increases the size of the orbital cavity, leading to the appearance of so-called ‘puppy dog eyes’. In domestic dogs, this expression was suggested to be enhanced by artificial selection and to play an important role in the dog-human relationship. Production of the inner brow raiser has been shown to be sensitive to the attentive stance of a human, suggesting a possible communicative function. However, it has not yet been examined whether it is sensitive to human presence. In the current study, we aimed to test whether the inner brow raiser differs depending on the presence or absence of an observer. We used two versions of a paradigm in an equivalent experimental setting in which dogs were trained to expect a reward; however, the presence/absence of a person in the test apparatus was varied. In the social context, a human facing the dog delivered the reward; in the non-social context, reward delivery was automatized. If the inner brow raiser has a communicative function and dogs adjust its expression to an audience, we expect it to be shown more frequently in the social context (when facing a person in the apparatus) than in the non-social context (when facing the apparatus without a person inside). The frequency of the inner brow raiser differed between the two contexts, but contrary to the prediction, it was shown more frequently in the non-social context. We further demonstrate that the inner brow raiser is strongly associated with eye movements and occurs independently in only 6% of cases. This result challenges the hypothesis that the inner brow raiser has a communicative function in dog-human interactions and suggests a lower-level explanation for its production, namely an association with eye movements.

## Introduction

Facial expressions accompany (putative) emotional states in humans and non-human animals (reviewed by Descovich et al., 2017) and can provide information about an individual’s intentions and potential future behavior (Waller et al., 2017), both in positive contexts such as signaling playful intent (Fox, 1970) and in negative contexts such as predicting aggression (Camerlink et al., 2018). While facial expressions have often been considered to be mainly reflexive and invariable, particularly when linked to emotional states (see e.g., Liebal et al., 2014; Scheider et al., 2016; reviewed by Jones et al., 1991; Kaminski et al., 2017), for humans and several non-human primate species there is evidence of *audience effects* on the production of facial expressions: individuals will adjust their facial displays depending on the presence or attentive state of an observer (e.g., Kraut and Johnston, 1979; Jones et al., 1991; Liebal et al., 2004; Poss et al., 2006; Demuru et al., 2015; Waller et al., 2015; Scheider et al., 2016). This sensitivity to an audience suggests a communicative function of the respective expression (Leavens et al., 1996), which may thus constitute a ‘*signal*,’ i.e., a behavior evolved for the purpose of information conveyance (Laidre and Johnstone, 2013). In contrast, a ‘*cue*’ constitutes a mere by-product of an animal’s behavior which may coincidentally convey information to another individual (Shariff and Tracy, 2011; Laidre and Johnstone, 2013). The only non-primate species where the effect of an audience on the production of facial expressions has so far been reported, to our knowledge, is the domestic dog (*Canis familiaris*) (Kaminski et al., 2017).

To assess whether human attention and/or an emotionally arousing stimulus affected facial expressions in dogs, Kaminski et al. (2017) compared dogs’ facial expressions directed at either an attentive person (standing in front of and facing the dog) or an inattentive person (turned away from the dog). Additionally, it was varied whether or not this person was holding a piece of food (considered to be an emotionally arousing stimulus) (Kaminski et al., 2017). In line with an audience effect, dogs’ facial expressions differed depending on the person’s attentive stance, and this effect was particularly strong for two actions: the ‘inner brow raiser’ and ‘tongue show,’ which were shown more often when the human was facing the dog than when she was turned away, implying a possible communicative function of these expressions (Kaminski et al., 2017). The visibility of the food item, however, did not significantly affect the dogs’ facial display, suggesting that it does not primarily constitute an emotional expression (Kaminski et al., 2017).

The inner brow raiser in particular has attracted researchers’ attention in the context of dog-human communication. By raising the medial part of the eyebrow, the inner brow raiser increases the height of the orbital cavity, thus creating the impression of larger eyes (Waller et al., 2013). This paedomorphic expression was hypothesized to be particularly attractive to humans (Waller et al., 2013). One study reported that in shelter dogs, the rate of the inner brow raiser (measured when a person was standing in front of the kennel) was inversely related to time at the shelter until rehoming (Waller et al., 2013). Dogs with a high frequency of raising the brow might thus have a selective advantage (Waller et al., 2013). This effect may not only be at work in the current environment, but by using rehoming speed as a proxy for human selection during evolution, it was proposed that performance of the inner brow raiser was selected for in dogs in the course of domestication (Waller et al., 2013).

To investigate this hypothesis further, Kaminski et al. (2019) compared the production of the inner brow raiser as well as anatomical features underlying this movement in dogs and their closest extant relatives, gray wolves (*Canis lupus*). The study indicated differences between the species in both anatomy and behavior: in dissections of six domestic dogs and four wolves, the muscle responsible for the inner brow raiser movement (*levator anguli oculi medialis* = LAOM) was typically pronounced in dogs, whereas in the wolves it was more variable, usually ill-defined and not a separate muscle (Kaminski et al., 2019). Kaminski et al. (2019) further compared the production of inner brow raiser movements in shelter dogs and captive gray wolves when a human observer was standing in front of the kennel/enclosure. A higher frequency and intensity of inner brow raiser movements were observed in the dogs compared to the wolves (Kaminski et al., 2019). Thus, Kaminski et al. (2019) concluded that artificial selection resulted in a change in the facial musculature of dogs to enhance dog-human communication.

If a behavior has a communicative function, it would be expected to vary contextually based on the presence or absence of a receiver of this expression. For example, chimpanzees were considered to use a behavioral action communicatively if it was shown more often when a human observer was present than in the absence of an audience (Leavens et al., 1996). However, this most basic form of an audience effect, namely whether dogs’ production of the inner brow raiser is affected by the presence of an audience (also referred to as social use, see Liebal et al., 2014; Waller et al., 2015), has not been tested so far.

Our first aim, therefore, was to investigate whether the inner brow raiser in dogs is sensitive to the presence of an audience. To this end, we compared dogs’ expression of the inner brow raiser in a social context with an interacting human and in a non-social context without face-to-face interaction with a human. Using a within-subjects design, dogs were trained to expect a reward from an apparatus where the reward was delivered either (1) through a remotely controlled reward-delivery system without a person inside the apparatus (non-social context) or (2) by a person sitting inside the apparatus and facing the dog (social context). The social context represented a situation in which dogs were expected to likely communicate with humans, namely when awaiting a reward to be delivered by a person (Gaunet, 2008, 2010). In addition, we varied other situational features and explored their effect on the inner brow raiser production to enhance the validity of our findings. Therefore, in both the non-social and the social context we also varied the valence of the trial (positive: anticipation of a reward; negative: prevention of access to a visible reward) and the reward type the dogs were conditioned to expect. We used food and toys as both are considered to function as rewards in dogs (Gerencsér et al., 2018). However, they can be associated with different appetitive behavioral actions (i.e., ingestion of a palatable item vs. object manipulation), motivational states (e.g., Burghardt et al., 2016), and individual responsiveness (Gerencsér et al., 2018). Based on the previous evidence that the inner brow raiser serves a communicative function (Kaminski et al., 2017), but does not reflect an emotional state (Caeiro et al., 2017; Kaminski et al., 2017; Bremhorst et al., 2019), we predicted a higher incidence of the inner brow raiser in the social context (when facing a human) than in the non-social context, but no effect of trial valence.

Our second aim concerned the proximate mechanisms of the inner brow raiser movement. We explored an alternative hypothesis for its production in different contexts, given that the principle of parsimony postulates that lower-level explanations have to be ruled out before drawing conclusions regarding cognitively more complex processes (see Epstein, 1984; Zentall, 2017). According to the manual on DogFACS (Waller et al., 2013), an anatomically based coding method to systematically identify facial appearance changes due to muscle movements in dogs, the inner brow raiser appears to accompany eye movements and can even be used to infer eye movements, which are sometimes hard to detect. However, if the inner brow raiser primarily accompanies eye movements, then differences in its production between contexts could be an artifact simply based on differences in gazing behavior, providing a possible lower-level explanation for observations of this facial expression. Empirical evidence for an association between the inner brow raiser and eye movements is lacking. Therefore, in a subsequent second step, we used the video samples generated for our first research aim to analyze the frequency of eye movements across the different contexts and their association with the inner brow raiser.

## Materials and Methods

### Subjects

Our subjects were 21 family pet dogs (12 females and 9 males; mean age: 4.76 years ± *SD* = 2.77; see Supplementary Table 1 for details), recruited personally or via social media. To minimize effects of morphological variation on the facial display, we included only one breed without morphological extremes, Labrador retrievers, and one Labrador cross with a Labrador-like morphology.

### Study Design

**FIGURE 1:**
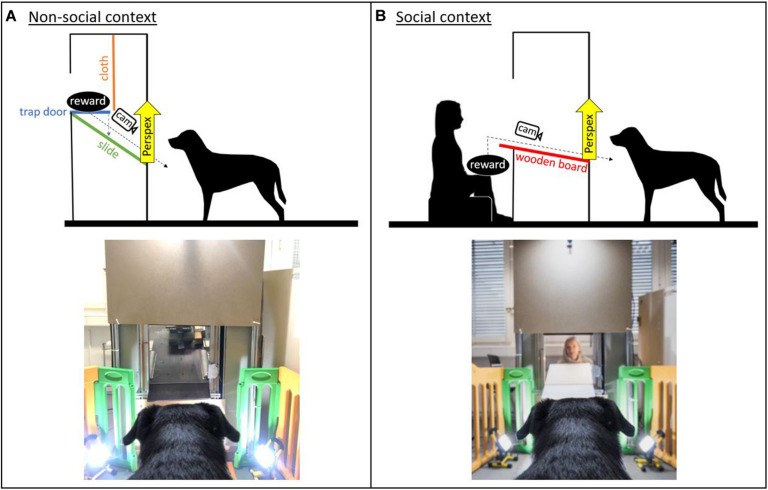
Sketch of the experimental set-up and image of the apparatus in the **(A)** non-social context and **(B)** social context (with the experimenter present inside the apparatus; image credit: Adrian Bear/Tierwelt).

The study consisted of two versions of a paradigm with an equivalent experimental setting and contingencies, except that a person was either absent (non-social context) or present (social context) inside a test apparatus (Figure 1). The dogs were conditioned to expect a desired reward (toy/food) to be delivered from this test apparatus. In the non-social context, the reward was delivered remotely. In contrast, in the social context, the experimenter was sitting inside the apparatus, visible to the dog, and handed the reward to the dog.

**FIGURE 2:**
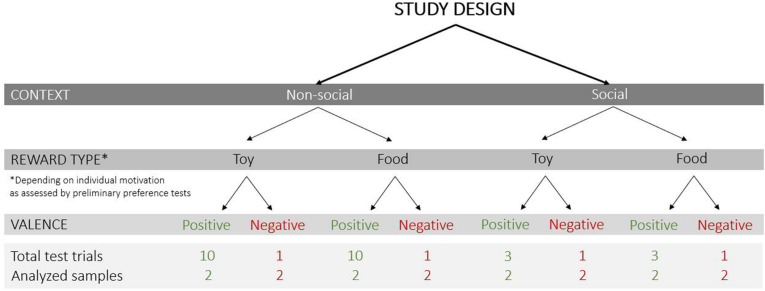
Study design with the test conditions that varied in the current study (context: non-social/social; reward type: toy/food; valence of the trial: positive/negative), the total number of test trials and analyzed samples (each sample was a 3 s video clip).

The following test conditions were varied (Figure 2): (1) context – non-social and social (absence or presence of a person inside the test apparatus), (2) reward type – toy and food, and (3) valence of the trial – positive (anticipation of access to a reward) and negative (prevention of access to a visible reward).

### Test Apparatus

The test apparatus was a custom-made wooden/metal construction (1.80 × 0.90 m) with a delivery window approximately at the dogs’ head height. The window could be covered using a remote-controlled transparent Perspex panel, which allowed for the filming of the dogs’ facial expressions while they were waiting for the reward. In the social context, a piece of cardboard was additionally used to cover the window to prevent the dogs from seeing the experimenter between trials. The interior of the apparatus varied between the non-social and the social context. In the non-social context, an automatic reward dispenser (functioning like a trap door) onto which the reward could be placed was mounted in the apparatus above the dog’s head height (Figure 1). The reward dispenser was hidden behind a piece of cloth to prevent the dogs from seeing the reward before it was delivered. In the social context, a wooden table was mounted in the apparatus and connected to the window. The experimenter sat in the apparatus so that her head was at approximately the same height as the reward dispenser in the non-social context (Figure 1).

### Experimental Procedure

#### Preliminary Preference Tests

With each dog, we conducted preference tests first between two toys and then between two food types, using paired presentations over 10 trials per reward type. As we only wanted to use rewards that the individual was motivated to obtain, the respective reward type was used for testing if the dog made a choice in at least eight trials, and the more frequently selected option was used in the subsequent procedure. All 21 dogs met this criterion with the food reward and 19 dogs with the toy reward. The 19 dogs that were sufficiently motivated for both reward types were additionally tested in a third preference test in which they could choose between their most preferred food and their most preferred toy over 10 trials. As all but two dogs preferred the food to the toy reward, this factor was not considered in the subsequent analyses.

#### Training

Training trials served to condition the dogs to approach the apparatus and to wait for 5 s until the reward was delivered. At the start of each trial, the window of the apparatus was covered by the Perspex panel and in the social context by the additional piece of cardboard. The owner was sitting on a chair 1.80 m from the apparatus with the dog next to her or him. The owner then released the dog and gave a verbal and visual release signal. In the first trials of the session (five trials in the first training session; in case a second training session was required, this was reduced to three), the owner then walked to the front of the apparatus and looked into it to draw the dog’s attention to this location. In all other trials, the owner remained sitting on the chair, which allowed us to see whether the dog approached the apparatus on her/his own, indicating the subject’s motivation and level of training to associate the apparatus with the reward. After 5 s, regardless of the dog’s behavior, the transparent panel was slid upwards by means of a remote-controlled system and the reward (which until then was out of the dog’s view) was delivered.

In the non-social context, reward delivery was performed by the automated system, i.e., as soon as the trap door was activated remotely, the reward fell onto a slide and slid down to the window, where it became accessible to the dog. In the social context, delivery was performed by the experimenter who handed the reward (which she had been holding in her hand below the wooden table) to the dog through the window. The dog could then consume the reward (ingest the food or play with the toy for a maximum of 30 s; this duration varied between individuals mainly due to differences in interest, play behavior, strength of motivation, obedience when returning the toy, etc.). At the end of each trial, the transparent panel was remotely activated to move down until it completely covered the window again. The next trial commenced shortly after the dog was back in the starting position next to the owner.

The training criterion to proceed to the test was that the dog immediately approached the apparatus on her or his own when released and waited in front of the apparatus until the reward was delivered in five consecutive trials. Only trials in which the owner remained sitting were evaluated for this purpose. This training criterion provided an objective means to evaluate the dog’s association between the apparatus and the reward and allowed to consider individual learning speed while keeping the number of repetitions as low as possible to avoid loss of interest.

A maximum of two training sessions with 10 trials each was conducted. If the dog did not reach the training criterion within these sessions, or if motivation decreased over repeated trials (i.e., the response deteriorated), training was terminated with this reward type in the respective context. The 19 dogs who were sufficiently toy motivated in the preference test were first trained with their preferred toy reward (and second with food) in both the non-social and the social context. Of these, 12 dogs reached the training criterion and were tested with the toy reward in the non-social context. In the social context, 15 dogs passed the training criterion and proceeded to testing with the toy (see Supplementary Table 1 for an overview). All 21 subjects were sufficiently motivated for the food reward in the preference test, reached the training criterion within two sessions in both the social and the non-social context and were therefore tested with food rewards in both contexts.

#### Testing

Positive and negative test trials were conducted (video examples of a positive and a negative test trial in the social and the non-social context are provided as Supplementary material). The procedure of the positive test trials was the same as in the training trials (described in section ‘Training’), with the 5 s delay until reward delivery considered as the ‘anticipation phase’. In the negative test trials, the reward was also delivered after 5 s, but the transparent panel did not open for 60 s (i.e., the ‘frustration phase’). During this time, the dog could see the reward lying in front of the transparent panel in the apparatus (non-social context), or in the experimenter’s hand (social context), but was unable to obtain it.

In trials of the social context (both training and testing), the experimenter always sought eye contact with the dog (without continuous direct staring) to facilitate a natural communicative interaction. The experimenter’s facial expression was friendly with a gentle smile to avoid any reluctance of the dogs to approach, which could be the case with a neutral face, as a neutral expression seems to be interpreted negatively by dogs (Racca et al., 2012; Ford et al., 2019).

All dogs first participated in the non-social context and subsequently in the social context. The fixed order of contexts was selected for reasons relating to project management and because we did not want to create an expectation of the experimenter handing the reward to the dog (as done in the social context) before the dog was tested in the non-social context. This might have attracted the dog’s focus away from the apparatus to the experimenter, who was also in the room during the non-social context (hidden behind a divider behind the dog) to operate the apparatus. Furthermore, the dogs always participated in the toy condition first, if applicable, as pilot studies had shown that loss of interest could be prevented by performing the session with the reward type that was preferred by nearly all subjects (food) after the session with the less preferred toy reward.

As a result of the fixed order of contexts, fewer training trials were required for the social context than for the non-social context, presumably because the dogs were already familiar with the procedure and the apparatus (mean number of evaluated trials until the training criterion was reached: non-social context—toy: 8.58, food: 5.33; social context—toy: 5.00, food: 5.00). Consequently, whereas in the non-social context training and testing of each reward type was performed in separate sessions to keep the number of repetitions low and prevent fatigue, in the social context training and testing could be combined in one session.

In the non-social context, five positive test trials were conducted before a single negative test trial. Five additional positive trials performed subsequently were aimed at reducing potential carry-over effects of this negative experience on the performance in the subsequent social context, although in the meantime we found that valence of the preceding trial does not seem to considerably affect expressions in the subsequent trial (Bremhorst et al., 2019). In the social context, two positive test trials were conducted directly after the training criterion was reached, followed by a single negative trial. A last positive test trial was aimed at ending the study with a positive experience for both the dog and the owner.

### Behavior Coding

#### Preparation of Video Samples

For each of the 21 subjects, two positive and two negative video samples of 3 s duration per reward type (food/toy when applicable) were created for each context (non-social/social). The duration of the samples was determined by the length of the positive trials; from the two positive trials directly preceding the negative trial, we used the middle 3 s from the ‘anticipation phase’ (i.e., ending 1 s before the transparent panel started to open). A previous study has shown that this time interval is long enough for several facial movements to occur (Bremhorst et al., 2019). For comparability, negative samples were of equal quantity and length as the positive samples, i.e., following the procedure of Bremhorst et al. (2019), two randomly selected negative samples of 3 s duration each were cut from the ‘frustration phase’ of the negative trial (excluding the first 10 s as the frustration response may not be triggered immediately).

A total of 276 samples was prepared, comprising 132 samples from the non-social context (toy positive: 24 samples, toy negative: 24 samples, both *N* = 12 (*N* refers to the number of subjects); food positive: 42 samples, food negative: 42 samples, both *N* = 21) and 144 samples from the social context (toy positive: 30 samples, toy negative: 30 samples, both *N* = 15; food positive: 42 samples, food negative: 42 samples, both *N* = 21).

#### Inner Brow Raiser Coding

**FIGURE 3:**
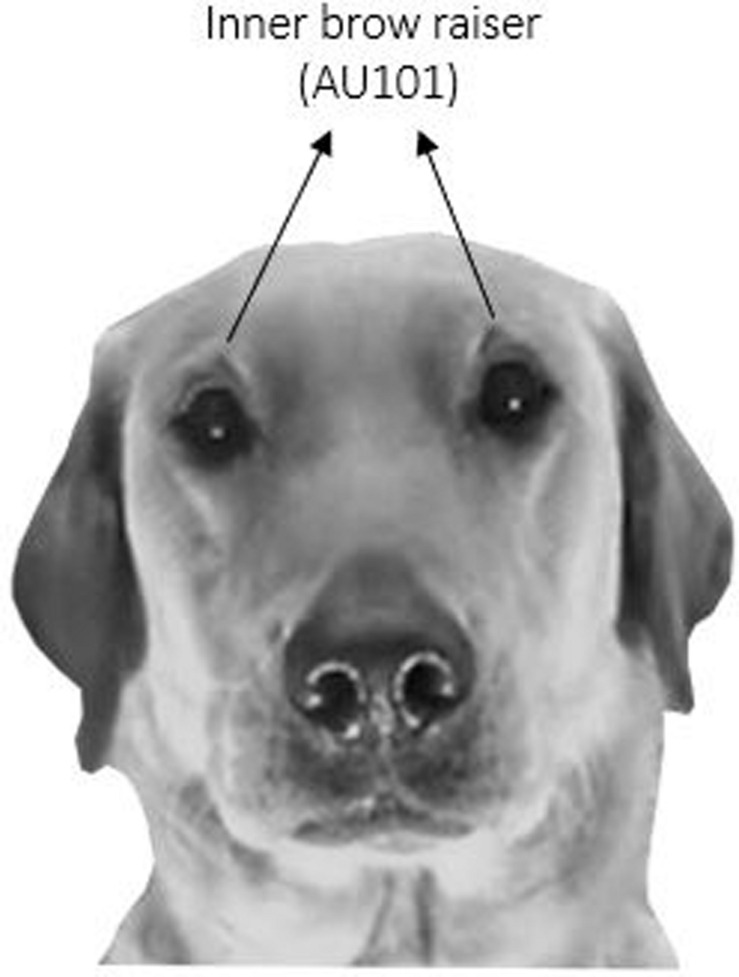
Dog producing a bilateral inner brow raiser movement.

Using DogFACS (Waller et al., 2013^1^), coding of the inner brow raiser (which is labeled with the code AU101) was performed (see Figure 3 for an example of a bilateral inner brow raiser). As a first step, the frequency of the inner brow raiser in the 276 samples was coded by two certified DogFACS coders, one of whom was blind to the research hypothesis. As is common practice to the authors’ knowledge, the inner brow raiser was coded independently of eye movements. Reliability between the coders over the 276 samples was very good with an average intraclass correlation coefficient of 0.80 (95% CI: 0.72–0.85).

#### Eye Movements and Combinations With the Inner Brow Raiser Coding

In a second step, we subsequently coded eye movements in four directions [left, right, up, down; as described in the DogFACS manual (Waller et al., 2013)]. To analyze the association between eye movements and the inner brow raiser, the following combinations of both behaviors were furthermore recorded: eye movements occurring (1) simultaneously (i.e., within 0.2 s) with the inner brow raiser (‘Eye movement present/inner brow raiser (*movement*) present’), (2) while the inner brow raiser remained tensed (‘Eye movement present/inner brow raiser (*tension*) present’), (3) without inner brow raiser movement or tension (‘Eye movement present/inner brow raiser absent’), or (4) inner brow raiser movement occurring without eye movement (‘Eye movement absent/inner brow raiser present’).

Coding was performed by a certified DogFACS coder who was blind to the research hypothesis, using a subsample of the original video samples. For this subsample, one positive and one negative sample per reward type from both the social and the non-social context were initially selected for each subject. We equally balanced between subjects whether the first or second of the two samples of each valence was used. However, if eye movement was hard to detect in the selected sample (mainly due to environmental conditions such as bad lighting or video quality such as insufficient sharpness), it was excluded from the analysis and the second sample of the corresponding condition was used if the eye movements were clearly detectable. It was not possible to obtain usable samples from all dogs from all conditions due to a lack of image quality; therefore the final subsample comprised 95 samples including 50 samples from the non-social context (toy positive: 10 samples, *N* = 10; toy negative: 11 samples, *N* = 11; food positive: 17 samples, *N* = 17; food negative: 12 samples, *N* = 12) and 45 samples from the social context (toy positive: 10 samples, *N* = 10; toy negative: 7 samples, *N* = 7; food positive: 14 samples, *N* = 14, food negative: 14 samples, *N* = 14). From each of the 21 individuals, at least one sample was included in the subsample.

To analyze intercoder reliability, a second certified DogFACS coder coded 20 of these samples (>20% of all videos of the subsample; 10 samples each were randomly selected from the social and the non-social context). Reliability between the two coders was very good with an average intraclass correlation coefficient of 0.93 (95% CI: 0.82–0.97) for ‘Eye movement present/inner brow raiser (*movement*) present’ and 0.89 (95% CI: 0.71–0.96) for ‘Eye movement present/inner brow raiser (*tension*) present’. There was a complete agreement for ‘Eye movement present/inner brow raiser absent’ and ‘Eye movement absent/inner brow raiser present’.

### Statistical Analyses

Statistical analyses were conducted in R Studio (version 1.2.1335).

#### Inner Brow Raiser

We analyzed whether the frequency of the inner brow raiser was affected by the test conditions that varied in the current study (context, reward type, valence of the trial) and by subject sex and age. Linear mixed effect models were computed (function: lme; package: nlme), using the frequency of the inner brow raiser as a response variable. Context (non-social/social), reward type (toy/food), valence of the trial (positive/negative), subject sex (female/male), and age were used as predictor variables. Subject ID was included as a random factor. Model assumptions were verified using visual inspection of the residuals.

To evaluate whether there was a relationship between the inner brow raiser and sample order within the social or the non-social context, we correlated the frequency of the inner brow raiser within each context with the sample number, using a repeated measures correlation (function: rmcorr; package: rmcorr; Bakdash and Marusich, 2017). When both reward types were tested within a context, the sample number ranged from one to eight; when only food was tested, it ranged from one to four.

#### Eye Movements and Combinations With the Inner Brow Raiser

To analyze whether the frequency of eye movements differed between the non-social and the social context and was affected by reward type, valence of the trial, subject sex, or age, linear mixed effect models were computed using the same approach as previously described for the inner brow raiser (section ‘Inner Brow Raiser’).

Associations between the inner brow raiser and eye movements were analyzed descriptively by comparing the frequencies of ‘Eye movement present/inner brow raiser (*movement*) present’, ‘Eye movement present/inner brow raiser (*tension*) present’, ‘Eye movement present/inner brow raiser absent’, and ‘Eye movement absent/inner brow raiser present’, and inferentially by computing a Cochran-Mantel-Haenszel chi-square test (function: cmh_test, package: coin). The four quadrants used for this test were the frequencies of events in which eye movements and/or the inner brow raiser were observed (‘Eye movement present/inner brow raiser present (*movement* and *tension* summarized)’, ‘Eye movement present/inner brow raiser absent’, ‘Eye movement absent/inner brow raiser present’) as well as ‘Eye movement absent/inner brow raiser absent’.

## Results

### Inner Brow Raiser

**FIGURE 4:**
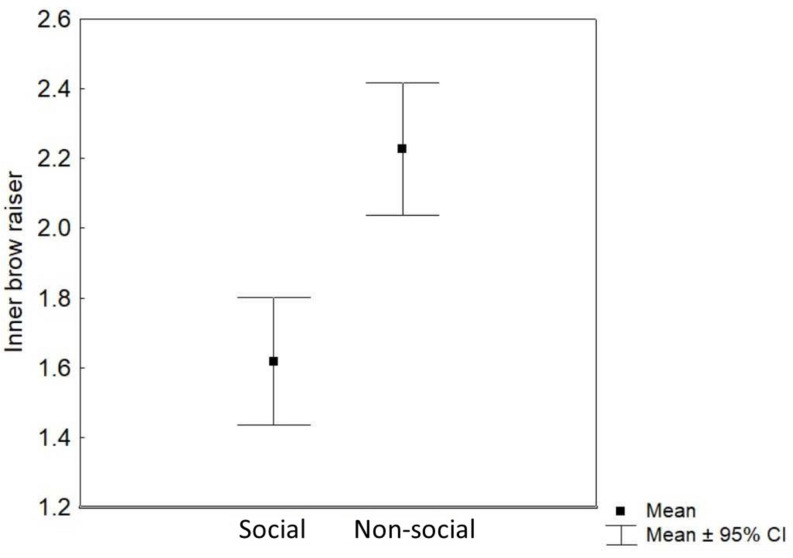
Mean and 95% confidence interval of the frequency of inner brow raiser movements per 3 s video sample in the social and the non-social context.

Context (non-social/social) was the only predictor that significantly affected the inner brow raiser production: the inner brow raiser was shown more frequently in the non-social context than in the social context [*F*_(1_, _252)_ = 24.62, *P* < 0.0001; *N* = 21; see Table 1 and Figure 4]. Neither reward type nor valence of the trial, subject sex, or age affected the frequency of the inner brow raiser significantly (Table 1).

**TABLE 1:** Results of the linear mixed effect model with the inner brow raiser as a response variable and context (social/non-social), reward type (toy/food), valence of the trial (positive/negative), subject sex (female/male), and age as predictor variables.

	Inner brow raiser			
Predictor	*df*	*F*	*P*	95% CI
**Context**	**1, 252**	**24.62**	**<0.0001**	**−0.89 to −0.39**
Reward type	1, 252	0.17	0.68	−0.22 to 0.31
Valence of the trial	1, 252	0.40	0.53	−0.33 to 0.17
Sex	1, 18	0.22	0.65	−0.59 to 0.28
Age	1, 18	0.92	0.35	−0.12 to 0.04

The frequency of the inner brow raiser was unrelated to sample order both within the non-social context (repeated measures correlation *r*_rm_ = 0.02; *P* = 0.87; 95% CI: −0.17 to 0.20; *N* = 21) and the social context (repeated measures correlation *r*_rm_ = -0.11; *P* = 0.23; 95% CI: −0.28 to 0.07; *N* = 21).

### Eye Movements and Combinations With the Inner Brow Raiser

As with the inner brow raiser, eye movements were significantly affected only by context: eye movements were produced more frequently in the non-social context than in the social context [*F*_(1_, _71)_ = 5.23, *P* = 0.03; *N* = 21]. There was no significant effect of reward type, trial valence, subject sex, or age (Table 2).

**TABLE 2:** Results of the linear mixed effect model with eye movements as a response variable and context (social/non-social), reward type (toy/food), valence of the trial (positive/negative), subject sex (female/male), and age as predictor variables.

	Eye movements			
Predictor	*df*	*F*	*P*	95% CI
**Context**	**1, 71**	**5.23**	**0.03**	**−1.24 to −0.10**
Reward type	1, 71	0.01	0.91	−0.58 to 0.61
Valence of the trial	1, 71	0.07	0.79	−0.49 to 0.62
Sex	1, 18	0.16	0.69	−0.59 to 0.90
Age	1, 18	0.004	0.95	−0.14 to 0.15

**FIGURE 5:**
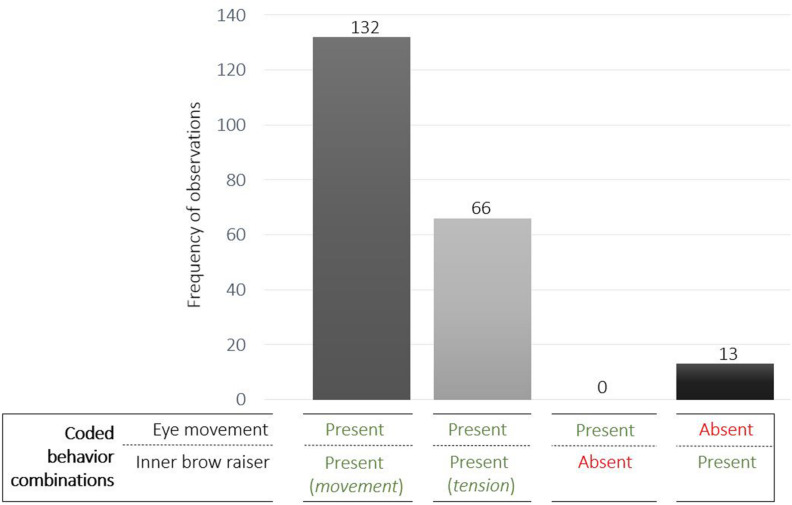
Frequency of observations of the coded behavior combinations of eye movements and/or the inner brow raiser.

Across all 211 observations of the inner brow raiser and/or eye movements, in 94% of cases (198 of 211 observations) eye movements occurred in conjunction with an inner brow raiser movement or inner brow raiser tension. In 63% (132 observations), the inner brow raiser movement was simultaneous with eye movements and in 31% (66 observations) the brows remained tensed while the eyes were moving (Figure 5). Eye movements were never observed without the inner brow raiser, and the inner brow raiser without eye movements was only observed in 6% of cases (13 observations; Figure 5).

The quadrant ‘Eye movement absent/inner brow raiser absent’ was calculated by first computing the maximum possible frequency of codable events in the subsample (consisting of 95 samples). In each sample (3 s duration), a maximum of 15 events could be coded (i.e., one event per observation unit of 0.2 s). From the resulting maximally codable 1,425 events in the subsample (i.e., 95 samples × 15 events), the frequencies of the coded events of each behavior combination were subtracted to obtain the frequency of events (0.2 s units) in which no eye movement or inner brow raiser was initiated (see Table 3). The association between the inner brow raiser and eye movements was highly significant (χ^2^_MH_ = 1322.1, *df* = 1, *P* < 0.0001; *N* = 21).

**TABLE 3:** 2 × 2 contingency table showing the four quadrants used for the Cochran-Mantel-Haenszel chi-square test, based on 95 video samples with 15 events each, resulting in a total of 1,425 events.

		Eye movement	
		Present	Absent
**Inner brow raiser**	**Present**	198 (*movement* + *tension*)	13
	**Absent**	0	1,214

## Discussion

Dogs’ expression of the inner brow raiser differed significantly between the non-social and the social context; however, contrary to the prediction, dogs performed the inner brow raiser more frequently in the non-social context, regardless of the expected reward type, trial valence, subject sex, or age. This direction of effect challenges the assumption that the inner brow raiser is used functionally by dogs for communication with humans (see Kaminski et al., 2017, 2019), and alternative explanations for the production of the inner brow raiser need to be considered.

Our results demonstrate that the inner brow raiser rarely occurs on its own but is usually shown in conjunction with eye movements. Likewise, eye movements were never observed without either the inner brow moving simultaneously or remaining tensed. Thus, the inner brow raiser appears to be an integral feature of eye movements. Consequently, the most likely explanation for the effects of the sociality of the context on the production of the inner brow raiser is the difference in gazing behavior between the social and the non-social context.

Several factors can potentially account for the lower frequency of gaze changes (and thus inner brow raiser movements) in the social context. As dogs are prone to looking at humans’ faces (Miklósi et al., 2003), in particular the eye area (Topál et al., 2014), the experimenter’s face was likely a highly salient stimulus for them to focus on. Furthermore, eye contact in a face-to-face setting, as it was the case in the social context, was described to increase dogs’ attention to a human’s face (Topál et al., 2014). Conversely, without a face to focus on, the dogs may have been looking around more in the non-social context. Importantly, as the experimenter was seated on a low stool in the current study, looking into her face (like looking at the automatic reward dispenser) did not require the dogs to move their eyes much – unlike in previous studies where the experimenters were standing (Waller et al., 2013; Kaminski et al., 2017, 2019) and the dogs would presumably have to look up to make eye-contact.

Another factor that could potentially have differed between the two contexts is the state of arousal. Arousal, which could be triggered by the proximity or orientation of another individual, has been considered a potential (lower-level) mechanism for audience effects (Zajonc, 1965; Liebal et al., 2014). In the current study, high arousal might be associated with greater vigilance and thus increased rates of eye movements and consequently brow movements. It could be hypothesized that dogs’ arousal declined over the course of the testing sessions (first the non-social context, then the social context) due to dogs habituating to the set-up and procedure. However, if arousal was driving the differences between contexts, we would also expect it to operate within each context, and the same should apply to arousal during the tests with different reward types (with the toy condition always preceding the food condition). The fact that there was no significant effect of reward type on the inner brow raiser argues against differential arousal levels as the decisive factor. Likewise, sample order did not have a significant effect on the production of the inner brow raiser. To better understand the effect of arousal on eye and inner brow movements, future studies could additionally collect physiological parameters that indicate a subject’s arousal level, such as heart rate (e.g., Zupan et al., 2016), eye or ear temperature (e.g., Riemer et al., 2016; Travain et al., 2016).

In the current study, we have demonstrated that the inner brow raiser is primarily incidental to eye movements in dogs and presumably not of general communicative value. The finding highlights the importance of considering simpler mechanisms before inferring cognitively more complex interpretations, as also recently discussed for the study of canine emotions (Zentall, 2017). We suggest that the previous findings on the possible social function of the inner brow raiser (Kaminski et al., 2017), might possibly also be explained by differences in gazing behavior. In the attentive condition of Kaminski et al. (2017), the human was standing 1 m from the dog. Hence, to look at the human’s face, the dogs would have to move their head and/or eyes upwards, which is less likely to have occurred in the inattentive condition, in which the human had her back turned to the dog. Thus, the increased production of the inner brow raiser could be an artifact of variation in gaze behavior between the two conditions.

The same explanation could potentially account for the observed differences in the production of the inner brow raiser reported in the comparative study with dogs and wolves (Kaminski et al., 2019). Dogs have been found to gaze more at humans’ faces than wolves (Miklósi et al., 2003; Gácsi et al., 2005); hence the increased frequency of the inner brow raiser shown by the dogs in Kaminski et al. (2019) would be consistent with the dogs looking at the experimenter’s face more often than the wolves. A study comparing captive wolves and dogs furthermore indicated that dogs are more alert during resting than wolves (Kortekaas and Kotrschal, 2019), which may also be associated with a higher likelihood of dogs responding to the human experimenter in the study by Kaminski et al. (2019). Moreover, the test conditions differed between species in Kaminski et al. (2019). Whereas the dogs were tested in kennels at an animal shelter, the wolves were tested in their home enclosure at an animal park. However, a person is likely to attract greater attention, and thus gazing, from shelter dogs, which are often relatively deprived of human contact, than from wolves at a wolf park. Besides, the wolves’ enclosures were likely larger than the dogs’ kennels, which would place the dogs closer to the human observer. This might have caused the dogs to look upwards more than the wolves, potentially leading to more accompanying brow movements. Dogs’ tendency to seek human proximity (e.g., Gácsi et al., 2001; Topál et al., 2005; Barrera et al., 2010) could have further increased this effect. These alternative lower-level explanations for the results of the previous studies remain speculative but seem to be consistent with all data now available. Future studies could test this hypothesis further by systematically varying the above-described conditions in both species under otherwise identical testing conditions to examine these suggested associations and further explore the importance of different factors influencing the occurrence of the inner brow raiser.

The fixed order of testing could be considered a potential limitation of the current study; however, we did not expect test order to considerably affect our findings, as a previous study with a similar methodology demonstrated no carry-over effects from previous trials on dogs’ facial expressions (Bremhorst et al., 2019), and likewise, no effect of trial number on the dogs’ facial expressions was reported in Kaminski et al. (2017). To test for potential order effects, we assessed the relationship between the inner brow raiser and sample order, which was non-significant. Furthermore, neither reward type (the toy condition always preceded the food condition) nor valence (the positive samples always preceded the negative samples) significantly affected the frequency of the inner brow raiser (see Table 1). These findings make it unlikely that our results can be explained by testing order.

To conclude, we propose a cognitively lower-level explanation for the differential occurrence of the inner brow raiser in dogs depending on the sociality of the context. Our work emphasizes the importance of considering alternative explanations for what might appear superficially to be functional behavioral expressions.

## Data Availability Statement

The original contributions presented in the study are included in the article/Supplementary Material, further inquiries can be directed to the corresponding author/s.

## Ethics Statement

The animal study was reviewed and approved by the cantonal authority for animal experimentation, the Veterinary Office of the Canton of Bern (Switzerland) (License no. BE62/18 30385) and the College of Science Research Ethics Committee, University of Lincoln (United Kingdom) (UID CoSREC304). Written informed consent was obtained from the owners for the participation of their animals in this study. Written informed consent was obtained from the individual(s) for the publication of any potentially identifiable images or data included in this article.

## Author Contributions

AB, SR, DM, and HW developed the study concept, design, and interpreted the data. AB conducted the experiments and drafted the initial manuscript. LS and AB (both certified DogFACS coders) coded the video samples using DogFACS. All authors reviewed and edited the manuscript.

## Conflict of Interest

The authors declare that the research was conducted in the absence of any commercial or financial relationships that could be construed as a potential conflict of interest.

## Footnotes

1 www.animalfacs.com
