# Supplementary material for: ‘Puppy Dog Eyes’ Are Associated With Eye Movements, Not Communication
Source: Front Psychol. 2021 Feb 17;12:568935. doi: 10.3389/fpsyg.2021.568935 (PMC7925631; doi:10.3389/fpsyg.2021.568935)
Supplement: Supplementary file 5 [file Data_Sheet_1.pdf]

Supplementary Material

1 Supplementary Table

Table 1. Details on the individual subjects tested in the social and non-social context with the toy and/or food reward. Ticks in the ‘Preference test’ columns indicate that the subject was sufficiently motivated for this reward type; ‘Preferred reward’ indicates the subject’s most preferred reward type when given the choice between the preferred toy vs. food reward. Ticks in the ‘Context’ columns indicate that subjects were tested with the respective reward type; crosses indicate that the training criterion was not reached or that training was ceased as motivation deteriorated during training.

ID	Breed	Sex (F=female; M=male)	Age (years)	Preference test			Context			
							Non-social		Social	
				Toy	Food	Preferred reward	Toy	Food	Toy	Food
1	Labrador	F	3.5	✓	✓	Food	✓	✓	✓	✓
2	Labrador	F	4	✓	✓	Food	✓	✓	✓	✓
3	Labrador	F	3	✓	✓	Food	✗	✓	✗	✓
4	Labrador	M	6.5	✓	✓	Food	✓	✓	✓	✓
5	Labrador	M	5.5	✓	✓	Food	✓	✓	✓	✓
6	Labrador	F	6.5	✓	✓	Food	✓	✓	✓	✓
7	Labrador	F	9.5	✗	✓	NA	✗	✓	✗	✓
8	Labrador	M	6.5	✗	✓	NA	✗	✓	✗	✓
9	Labrador	M	3.5	✓	✓	Food	✗	✓	✓	✓
10	Labrador cross	F	8	✓	✓	Toy	✓	✓	✓	✓
11	Labrador	F	4.5	✓	✓	Food	✗	✓	✗	✓
12	Labrador	M	3.5	✓	✓	Toy	✓	✓	✓	✓
13	Labrador	F	2	✓	✓	Food	✓	✓	✓	✓
14	Labrador	M	1	✓	✓	Food	✓	✓	✓	✓
15	Labrador	M	3	✓	✓	Food	✓	✓	✓	✓
16	Labrador	M	4	✓	✓	Food	✓	✓	✗	✓
17	Labrador	F	1.5	✓	✓	Food	✗	✓	✓	✓
18	Labrador	F	3	✓	✓	Food	✗	✓	✓	✓
19	Labrador	F	6.5	✓	✓	Food	✗	✓	✓	✓
20	Labrador	F	12.5	✓	✓	Food	✗	✓	✗	✓
21	Labrador	M	2	✓	✓	Food	✓	✓	✓	✓
